# Vegetation types shape the soil micro-food web compositions and soil multifunctionality in Loess Plateau

**DOI:** 10.3389/fmicb.2025.1523811

**Published:** 2025-02-12

**Authors:** Zhiming Chen, Wenjuan Kang, Renyuan He, Guang Li, Zhuzhu Luo

**Affiliations:** ^1^College of Forestry, Gansu Agricultural University, Lanzhou, China; ^2^Key Laboratory of Grassland Ecosystem (Gansu Agricultural University), Ministry of Education, Lanzhou, China; ^3^College of Resources and Environmental Sciences, Gansu Agricultural University, Lanzhou, China

**Keywords:** Loess hilly area, multifunctionality, soil micro-food web, soil nematode, vegetation type

## Abstract

**Introduction:**

Vegetation degradation and soil erosion are severe problems in the Loess hilly region, rendering it one of the most ecologically vulnerable areas in China and globally. Vegetation restoration has been recognized as an effective approach to amending the fragile ecological environment and restoring degraded ecosystems.

**Methods:**

The effects of different vegetation types: *Caragana korshinskii, Prunus armeniaca L., Pinus tabuliformis* Carrière, *Medicago sativa* L., and the control vegetation Stipa bungeana on soil micro-food webs and soil multifunctionality, as well as their response mechanisms to soil environmental drivers, were investigated using High-throughput sequencing technology.

**Results:**

*C. korshinskii* significantly enhanced soil physicochemical properties and soil enzyme activities by facilitating the stability of the soil micro-food web structure driven by soil bacteria and fungi and increasing the soil multifunctionality in contrast to *S. bungeana*. *Prunus armeniaca* also improved soil multifunctionality by promoting soil organic carbon and alkaline phosphatase activity. However, the stability of the soil micro-food web structure and soil multifunctionality were suboptimal in *P. tabuliformis* and *M. sativa*. Soil pH, along with carbon, nitrogen, and phosphorus cycling nutrients and enzymes, profoundly influences the structure of the soil micro-food web and soil multifunctionality; among these factors, those related to the carbon and phosphorus cycles are identified as key influencing factors.

**Discussion:**

Therefore, a vegetation restoration strategy prioritizing *C. korshinskii* as the dominant vegetation type, supplemented by *P. armeniaca*, significantly impacts restoring soil multifunctionality and stabilizing the soil micro-food web in Loess hill regions and comparable ecological areas.

## Introduction

Loess Hill constitutes a distinctive geomorphic characteristic in the Loess Plateau of China and other appropriate regions globally (Deng et al., 2015a). The Longzhong Loess hilly region is positioned within the arid and semiarid zone of the Loess Plateau in northern China (Lu et al., 2014; Wang et al., 2017). The loose structure, along with rainfall predominantly manifesting in the form of heavy rain from July to September (Zhang et al., 2012; Sun et al., 2016; Zhang B. Q. et al., 2016), has engendered severe soil erosion and a scarcity of water resources in this region (Zhao et al., 2013; Gao et al., 2014; Li B. B. et al., 2021). Consequently, soil multifunctionality decreases. Soil multifunctionality stems from the interaction between many soil biological communities and the soil environment. This indicates that soil can provide nutrients, maintain nutrient cycling, store nutrients, decompose organic matter, and conduct other essential ecological functions and services. As a result, it serves as a crucial indicator of soil ecosystem health and an important parameter for studying ecosystem multifunctionality. The capacity of soil multifunctionality is significantly influenced by the structure of the soil micro-food web and its associated biodiversity (Delgado-Baquerizo et al., 2017). For example, soil microorganisms and nematodes influence soil multifunctionality by decomposing litter from various vegetation types and regulating critical ecological processes, such as soil biological and chemical cycling (Wardle et al., 2004; Wan et al., 2022; Li et al., 2022a).

The soil micro-food web is a complex non-linear system (Deng et al., 2013) with diverse structures and functions (Yu et al., 2016; Guo et al., 2018). It is crucial in connecting aboveground and subsurface ecological processes (Zhao et al., 2014), influencing the nutrient flow and material circulation of terrestrial ecosystems, and serving as the basis of soil ecological functions. As such, it has become an integral focus of research on global terrestrial ecosystems and soil multifunctionality (Wan et al., 2022; Zhu et al., 2023). The key positions of the soil micro-food web are frequently occupied by soil nematodes and soil microorganisms (Yeates et al., 1993; Hu et al., 2024). Soil nematodes are widely distributed in various habitats and occupy numerous nutrient levels of the soil micro-food web (Wang et al., 2014; Zhang et al., 2024). The community characteristics of soil nematodes can effectively reflect the structure and function of the soil micro-food web, rendering them an important indicator of ecosystem restoration processes (Kudrin et al., 2021; Zhou, 2022). Additionally, soil nematodes selectively prey on soil microorganisms to sustain their growth and development (Gong et al., 2023). This predation stimulates the activity of soil microorganisms, affects microbial biomass and its metabolic activities (Ji et al., 2023), and eventually regulates the structure of the soil micro-food web (Bongers and Ferris, 1999; Zhou et al., 2021). In turn, soil microorganisms can interact with soil nematodes, influencing the function of the soil ecosystem (Albornoz et al., 2022).

Vegetation types profoundly affect the evolution of soil nematode communities and the structure and function of soil micro-food webs, modifying soil multifunctionality (Wagner et al., 2015; Zhang A. L. et al., 2021). In 1999, the Chinese government launched the “Grain for Green Project” (Deng et al., 2015b) to restore the ecological environment of the soil. Since its commencement, this project has significantly improved vegetation coverage within the Loess hilly area (Jia et al., 2019). Consequently, distinct arbors, shrubs, and grassland vegetation have thrived (Chen et al., 2015). The soil of *Stipa bungeana* demonstrated a high root density and turnover rate. In contrast, as the number of planting years increases, *Caragana korshinskii* will accumulate more litter and undergo more substantial root death, thereby facilitating soil organic matter accumulation (Rasse et al., 2005). The litter from the arbor forest *Pinus tabuliformis* contains many recalcitrant compounds, which can lead to a decline in soil fertility and impede the growth of other vegetation. Particularly in Loess hilly areas with poor soil, planting *P. tabuliformis* further exacerbates the deterioration of the ecological environment (Yang et al., 2010; Ali et al., 2019). Different vegetation types influence the quantity of organic matter and microbial biomass entering the soil through their diverse plant characteristics, surface vegetation coverage, distribution patterns, and community structure (Fu et al., 2017; Hernández-Cáceres et al., 2022), thereby modifying the soil structure and nutrients (Loch et al., 2000). Consequently, the soil micro-food web exhibits distinct response mechanisms to different vegetation types (Rajasekaran et al., 2014; Xiao et al., 2022; Hu et al., 2023).

In summary, establishing appropriate plant communities in fragile ecological environments like the Loess Plateau can optimize the soil micro-food web structure, thereby effectively enhancing soil multifunctionality and improving the ecological environment. Nevertheless, the majority of the current studies in this region primarily focus on the impacts of individual vegetation types on soil physical and chemical properties (Wilschut and Geisen, 2020), as well as the evolution of soil nematodes and microbial communities (Sun et al., 2018). Scant comprehensive research cases have investigated the response mechanisms of soil micro-food webs and soil multifunctionality to different vegetation types.

In this study, the Anjiagou Basin in Anding District, Dingxi City, Gansu Province, in the Longzhong Loess hilly region, was selected as the research area. The typical artificial vegetation types of arbors, shrubs, and grasslands were chosen as the research objects. This study used high-throughput sequencing technology to investigate the structure and diversity of soil nematodes and microbial communities across different artificial vegetation types within the study areas. Structural equation models were then established to elucidate the response mechanisms of the soil micro-food web and soil multifunctionality to other vegetation types. We hypothesized that different vegetation types modify soil physicochemical properties, influence microbial and nematode community characteristics, and consequently modulate the structure of micro-food webs and soil multifunctionality. The findings of this study are anticipated to provide a theoretical foundation for the ecological restoration of the Loess Plateau and to serve as a valuable reference for similar regions globally.

## Materials and methods

### General situation of the study area

The experimental site was located in the experimental monitoring area (34°26′-35°35′N, 103°52′-105°13′E) of Dingxi Research Institute of Soil and Water Conservation, Gansu Province, in the Loess Plateau in northwest China. It pertains to the typical V subregion of the semiarid loess hilly and gully area. The soil type is yellow meadow, with weak erosion resistance and low organic matter content. The study area is characterized by a temperate continental monsoon climate, with an average altitude of 1,900–2,250 m. The mean annual temperature was 6.3°C, and the average annual precipitation was 427 mm, while evaporation reached approximately 1,500 mm. Precipitation is concentrated in summer, primarily occurring as heavy rainfall events.

Before 1999, a significant portion of agricultural land in the study area was abandoned and naturally succeeded in *S. bungeana* grassland, forming a natural restoration ecosystem that has remained in a state of natural succession. In 1999, on this grassland, *Prunus armeniaca* L. forest, *P. tabuliformis* forest, and *C. korshinskii* shrublands were individually planted as artificial vegetation. In 2001, *Medicago sativa* L. was planted on the existing *S. bungeana* grassland. With *S. bungeana* as the control, sampling points were set up in the planting areas of *P. armeniaca*, *P. tabuliformis*, *C. korshinskii*, and *M. sativa* in July 2023 to study the effects of vegetation types on soil micro-food web structure and soil multifunctionality.

### Sample collection

Soil samples were collected from the rhizosphere soil at a 0–30 cm depth using the five-point sampling method in July 2023, during the peak growth season of artificial vegetation in the Loess hilly area of Longzhong. Four soil samples were obtained from each point, and one composite sample was formed by blending the four individual samples. Each treatment plot had four replicates, and the sampling area was 7 × 7 m. After eliminating impurities from the soil samples, such as stones, gravel, and plant residues, they were thoroughly mixed to form a homogeneous composite sample. The sample was then passed through a 2 mm sieve and promptly preserved on ice. Each soil sample was partitioned into two aliquots for analysis. The first aliquot was placed in a sterilized centrifuge tube, immediately transferred to a foam box containing ice packs, and promptly transported to the laboratory. Then, the aliquot was stored in a −80°C refrigerator to extract the total soil DNA. The second aliquot was placed in a dedicated aluminum box to determine the soil moisture content. The residual soil samples were transported back to the laboratory and air-dried in a shaded area to measure the physical and chemical parameters of the soil.

### Determination of soil physicochemical properties and enzyme activity

A total of 10 soil physical and chemical parameters were determined: soil moisture, electrical conductivity, pH, soil organic carbon, total nitrogen, nitrate nitrogen, ammonium nitrogen, total phosphorus, available phosphorus, and available potassium.

Soil moisture was quantified using the oven drying approach at a temperature of (105°C ± 2°C). Electrical conductivity was measured with the METTLER TOLEDO FE38 benchtop conductivity meter. The pH value was ascertained using the glass electrode method. Soil organic carbon was determined using the chromic acid oxidation heating technique (Sanmanee and Suwannaoin, 2009). Total nitrogen was determined using the Kjeldahl nitrogen determination method. Nitrate and ammonium nitrogen were assayed by the colorimetric method in a continuous flow analyzer (Brookes et al., 1985). Total phosphorus was determined using the molybdenum-antimony dichromate colorimetric method (Levine et al., 1955; Li B. B. et al., 2021). NaHCO_3_ extraction colorimetric method was used for available phosphorus (Bao, 1998). The available potassium was determined using the CH_3_COONH_4_ extraction-flame photometry method (Bao, 1998).

Four extracellular enzymes and one polyphenol oxidase associated with the soil carbon, nitrogen, and phosphorus cycles were analyzed. Glucosidase (β-1,4-glucosidase) and sucrase, both related to the soil carbon cycle, were quantified using p-nitrophenol colorimetry and 3,5-dinitrosalicylic acid colorimetry (Bell et al., 2013). Urease, which is linked to the soil nitrogen cycle, was measured using indophenol blue colorimetry (Tian et al., 2017; Jiao et al., 2018). Alkaline phosphatases connected to the soil phosphorus cycle were assessed using alkaline phosphonodisodium phosphate colorimetry (Li H. Z. et al., 2021). Polyphenol oxidase activity was determined using pyrogallol colorimetry (Ghiloufi et al., 2019).

### Calculation of soil multifunctionality

Soil multifunctionality was assessed using the following variables: (1) soil environmental factors (soil moisture, electrical conductivity, and pH), (2) carbon cycling nutrients (soil organic carbon), (3) nitrogen cycling nutrients (total nitrogen, nitrate nitrogen, and ammonium nitrogen), (4) phosphorus cycling nutrients (total phosphorus and available phosphorus), (5) soil carbon cycling enzymes (ß-1,4-glucosidase and sucrase), nitrogen cycling enzyme (urease), and phosphorus cycling enzyme (alkaline phosphatases). The average method was used in this process, yielding similar results to the multi-threshold method (Jing et al., 2015). Each variable was normalized using a Z-score transformation, after which the versatility index was calculated by averaging the normalization rates of the variables (Ma et al., 2022). The Z-score conversion of variables was performed using SPSS version 19.0.

### Soil DNA extraction and high-throughput sequencing

Soil DNA was extracted from a 0.5 g soil sample using the E.Z.N.A Soil kit (Omega Bio-tek, Norcross, GA, United States). The concentration and purity of the extracted DNA were evaluated with a NanoDrop 2000 UV-VIS spectrophotometer (Thermo Scientific, Wilmington, United States), and the quality of the extraction was assessed through 1% agarose gel electrophoresis (Porazinska et al., 2010; Tian, 2022).

Primers NF1 (5′-GGTGGTGCATGGCCGTTCTTAGTT-3′) and 18S r2bR (5′-TACAAA GGGCAGGGACGTAAT-3′) (Feng et al., 2017) were employed to amplify the V4 segment of soil nematode DNA (Du et al., 2020) under the following conditions: 95°C pre-denaturation for 3 min; 95°C denaturation for 30 s, 55°C annealing for 30 s, 72°C elongation for 45 s, for 35 cycles; 72°C elongation for 10 min, and storage at 4°C. For bacteria, the V3-V4 region of the 16S rRNA gene was amplified using the primers 515F (5′-GTGCCAGCMGCCGCGG-3′) and 907R (5′-CCGTCAATTCMTTTRAGTTT-3′) (Wang and Wang, 1996). The amplification conditions were as follows: pre-denaturation at 98°C for 2 min; denaturation at 98°C for 15 s, annealing at 55°C for 30 s, extension at 72°C for 30 s, final extension at 72°C for 5 min, with 30 cycles. For fungi, the ITS1 region was amplified using the primers ITS1F (5′-CTTGGTCATTTAGAGGAAGTAA-3′) and ITS1R (5′-GCTGCGTTCTTCATCGATGC-3′) (Yang et al., 2017). The amplification conditions were as follows: pre-denaturation at 95°C for 5 min, denaturation at 95°C for 1 min, annealing at 50°C for 1 s and extension at 72°C for 1 min, and final extension at 72°C for 7 min, with 15 cycles. PCR was conducted by employing Trans Start Fastpfu DNA Polymerase (Trans Gen AP221-02) on a PCR instrument (ABI Gene Amp^®^ 9700 type) (Fan et al., 2023).

The procedure was repeated more than three times for a single sample, and the PCR products obtained from the same sample were uniformly mixed and analyzed using 2% agarose gel electrophoresis. The PCR products were recovered utilizing the Axy Prep DNA Gel Recovery Kit (AXYGEN, United States), eluted with Tris-HCl, and re-evaluated through 2% agarose gel electrophoresis. Quantitative detection of the PCR products was conducted using the Quanti Fluor-ST Blue fluorescence quantification system (Promega, Beijing, China). Following the sequencing volume requirements for each sample, the PCR products were combined in appropriate proportions. The MiSeq library was constructed using polymerized DNA products and sequenced on Illumina MiSeq PE300, a high-throughput sequencing platform (performed by Shanghai Meiji Biomedical Technology Co., Ltd.). The resulting high-throughput sequencing data were subsequently utilized for further data analysis.

### Data analysis

#### Analysis of soil nematodes and soil microbial diversity

The α diversity of microbial and nematode communities was evaluated using the Shannon index (Shannon, 1997). A non-metric multidimensional scaling analysis was performed to investigate differences in community structure among treatments at the OUT level, specifically, beta diversity (Shen et al., 2022), using the vegan package in R software version 4.4.0. The Bray-Curtis algorithm was adopted to calculate the sample distances, and the ANOSIM test was utilized to assess the significance of community structure changes (*P* < 0.05). A stress value < 0.2 indicates a meaningful graph interpretation. The data on the diversity and structure of soil microbial and nematode communities were obtained from the I-Sanger cloud platform of the Shanghai Meiji Company.

#### The ecological function index of the soil nematode community

The micro-food web structure, nutrient enrichment conditions, and decomposition pathways of the soil ecosystem were assessed using various nematode-based indicators (Ferris and Bongers, 2006). These indicators include the basic index and maturity index of free-living nematodes (Bongers, 1990), the plant-parasitic nematode maturity index (Bongers, 1990), channel index, structural index (SI), and enrichment index (EI) (Ferris et al., 2001; Ferris and Matute, 2003).

#### The metabolic footprint of the soil nematode community

The metabolic footprint of nematodes (NMF) was determined by calculating the fresh weight of nematodes, as outlined in the Nematodes - Plant Expert Information System.


NMF=Σ[((N×t(0.1×(W÷tm)t+0.273(W)t0.75)))]

where N_t_ is 1. The abundance of nematodes affiliated with trophic group t, M_t_ is the c-p value of them, and W_t_ is the biomass (Ferris, 2010).

The metabolic footprint of nematode nutrient groups includes the metabolic footprint of bacterial feeders, fungal feeders, plant-parasites, and omnivores-predators. The first three categories represent the carbon and energy input from bacteria, fungi, and plants into the food web. In contrast, the latter category indicates the carbon and energy input into the high trophic levels of omnivorous-predatory nematode populations. The total nematode metabolic footprint quantifies the overall metabolic footprint of nematode populations (Ferris, 2010).

#### Soil nematode faunal analysis

The enrichment metabolic footprint (*Fe*) and structural metabolic footprint (*Fs*) were utilized to analyze the nematode fauna. The enrichment footprint (*Fe*) represents the metabolic footprint of nematode populations featuring low c-p values (1–2), which can rapidly respond to resource accumulation. The structure footprint (*Fs*) reflects the metabolic footprint of nematodes with a high c-p value (3–5) (Ferris, 2010; Chang et al., 2021). By taking the coordinate point (SI, EI) as the central position, delineate and determine the coordinate positions (SI–0.5*Fs*/k, EI), (SI + 0.5*Fs*/k, EI), (SI, EI–0.5*Fe*/k), (SI, EI + 0.5*Fe*/k) of each treatment in four quadrants. Where k denotes the conversion factor (Zhang et al., 2015).

#### Analysis of energy flow in the soil micro-food web

The energy flow analysis of the soil micro-food web was conducted using the method described by Ferris and Bongers (2006). The coordinates of the vertex are (50, 86.6), while the coordinates of the lower left and lower right corners of the triangle are (0, 0) and (100, 0), respectively. The coordinates for each treatment were calculated based on the relative metabolic footprints of bacterial feeders, fungal feeders, and plant–parasitic nematodes, and a scatter plot was generated.

#### Correlation network analysis

A correlation network analysis evaluated the relationships between microorganisms and nematodes. We focused on the relative abundances at the genus level, excluding interactions between genera within the same species to simplify these correlations. We identified all Spearman correlations that appeared in at least three samples. Intergeneric co-occurrences were considered valid if the Spearman correlation coefficient (r) exceeded 0.6 and the *p*-value was less than 0.05 (Deng et al., 2012). Network visualization was performed using Gephi v0.9.2.

#### Structural equation model

IBM SPSS Amos 28 was utilized to conduct path analysis, revealing the relationships between nematode metabolic footprint, microbial community, and soil organic carbon (Arbuckle, 2006). The optimal model was determined through the iterative elimination of non-essential paths. Arrows and their associated path coefficients indicate the direction and strength of the relationships among these variables. Model fitness was evaluated using several indices: the chi-square (χ^2^) statistic and its corresponding *p*-value, the comparative fit index (CFI), goodness-of-fit index (GFI), root mean square error of approximation (RMSEA), normalized fit index (NFI), and Tucker-Lewis index (TLI).

Furthermore, a structural equation model was constructed using IBM SPSS Amos 28 to elucidate the potential relationships among soil physicochemical properties, enzymes, micro-food webs, and soil multifunctionality. Before modeling, principal component analysis was conducted on four groups of variables, excluding single indices: (1) soil environmental factors (soil moisture, conductivity, and pH), (2) nitrogen cycling nutrients (total nitrogen, nitrate nitrogen, and ammonium nitrogen), (3) phosphorus cycling nutrients (total phosphorus and available phosphorus), and (4) soil carbon cycling enzymes (β-1,4-glucosidase and sucrase). The first principal component from each group was then extracted for modeling (Liu et al., 2024).

#### Mantel test, random forest, and redundancy analysis

The correlation between soil microbial and nematode communities, soil physicochemical properties, and soil enzymes was assessed using the Mantel test (Liu et al., 2024). Random forest analysis revealed the influence of soil physicochemical properties and enzymes on various trophic groups of nematodes, soil microorganisms, and soil multifunctionality. The Mantel test and random forest analyses were conducted using R version 4.4.0 (Liu et al., 2024). Redundancy analysis was performed to examine the relationships between nematode and microbial communities and soil environmental factors using CANOCO 5.0.

Statistical analyses and data visualization were conducted using SPSS 19.0, Prism 8.0.2, and Origin 2024. A one-way analysis of variance (ANOVA) was performed, followed by multiple comparisons using the least significant difference (LSD) method (*p* = 0.05). Data are presented as mean ± standard error.

## Results

### Diversity and community structure of soil microorganisms and nematodes

**FIGURE 1:**
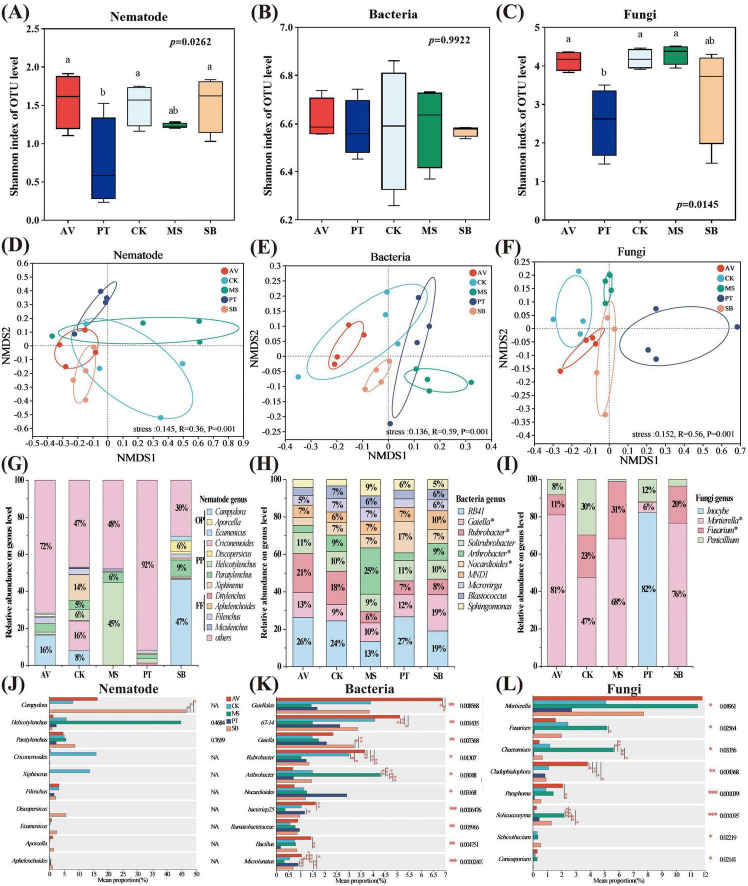
Alpha **(A–C)** and beta **(D–F)** diversity and structure characteristics of soil microorganism and nematode communities under different vegetation types. Different lowercase letters indicate significant differences at *P* < 0.05 based on one-way ANOVA. AV, *Prunus armeniaca* L.; PT, *Pinus tabuliformis* Carrière; CK, *Caragana korshinskii*; MS, *Medicago sativa* L.; SB, *Stipa bungeana*. The horizontal and vertical coordinates denote the relative distances. **(G–I)** The relative abundance of soil microorganisms and nematode communities (greater than 1%) under different vegetation types. OP, omnivorous-predatory nematodes; PP, plant parasitic nematodes; FF, fungi-feeding nematodes. The asterisk (*) indicates a significant difference between treatments (*P* < 0.05). **(J–L)** Significance test analyses of the differences in dominant genera of soil microorganisms and nematode communities between different vegetation types based on one-way ANOVA analysis. *P ≤ 0.05, **P ≤ 0.01, ***P ≤ 0.001.

Significant differences were observed in the Shannon indices of nematode and fungal communities across different vegetation types (*P* < 0.05). Specifically, compared to *P. tabuliformis*, the Shannon indices of nematode and fungal communities in *P. armeniaca* increased by 113 and 62%, respectively. In comparison, those in *C. korshinskii* increased by 106 and 63%, respectively (Figures 1A–C). Moreover, vegetation type significantly influenced the community composition of soil microorganisms and nematodes (*P* = 0.001, stress < 0.15) (Figures 1D–F). In terms of relative abundance, omnivorous-predatory nematodes were most abundant in *S. bungeana* (relative abundance > 77%), whereas plant-parasitic nematodes predominated in other vegetation types (relative abundance > 72%) (Figure 1G). In *M. sativa*, the bacterial genus *Arthrobacter* exhibited the highest relative abundance (25%), whereas RB41 showed the highest relative abundance in all other treatments (> 19%) (Figure 1H). Among fungal genera, *Inocybe* exhibited the highest relative abundance in *P. tabuliformis* (82%), whereas *Mortierella* had the highest relative abundance in all other treatments (> 47%) (Figure 1I). Compared with *S. bungeana*, at the genus level of nematodes (Figure 1J), the proportion of *Campydora* in *P. tabuliformis*, *M. sativa*, and *C. korshinskii* treatments was significantly lower (*P* < 0.05); at the bacterial genus level (Figures 1K, H), the proportion of *Microlunatus* and *Rubrobacter* in *P. armeniaca* and *Arthrobacter* in *M. sativa* were significantly higher (*P* < 0.05), while the proportion of *Microlunatus* in *M. sativa* was significantly low (*P* < 0.05). Additionally, the proportion of *Gaiella* in *M. sativa* and *C. korshinskii* was significantly low (*P* < 0.05). At the fungal genus level (Figures 1I, L), the proportion of *Chaetomium* in *M. sativa* and *Cladophialophora* in *P. armeniaca* showed a significant increase compared to that in *S. bungeana* (*P* < 0.05) (Figures 1J, L).

### Interrelationships within the soil micro-food web

**FIGURE 2:**
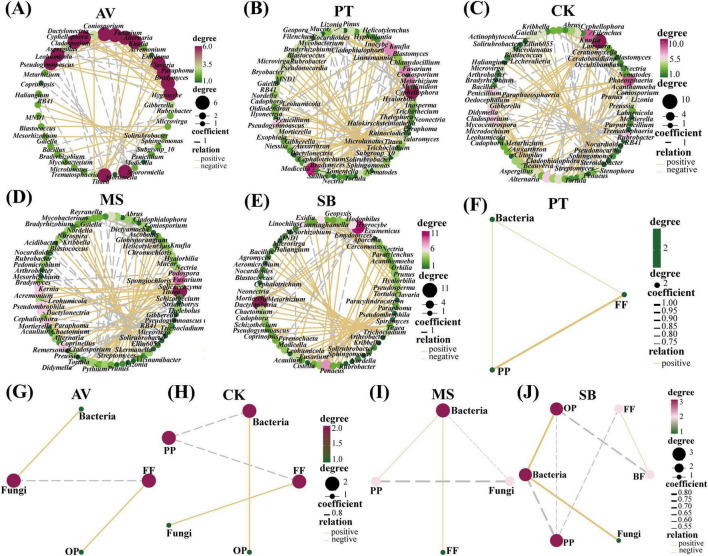
Correlation network diagram of the interaction intensity within the soil micro-food web under different vegetation types. The size of each node is proportionate to its centrality, and genera with higher centrality represent the keystone species of each network. The lines between nodes signify strong positive (yellow) or negative (dashed gray) interactions, and the thickness of the lines indicates the intensity of these correlations. **(A–E)** The interaction intensity among the top 50 most abundant bacterial, fungal, and nematode genera in the soil micro-food web under five distinct vegetation types. **(F–J)** The interaction intensity between bacteria, fungi, and nematodes with different feeding characteristics in the soil micro-food web under five distinct vegetation types. AV, *Prunus armeniaca* L.; PT, *Pinus tabuliformis* Carrière; CK, *Caragana korshinskii*; MS, *Medicago sativa* L.; SB, *Stipa bungeana*.

**FIGURE 3:**
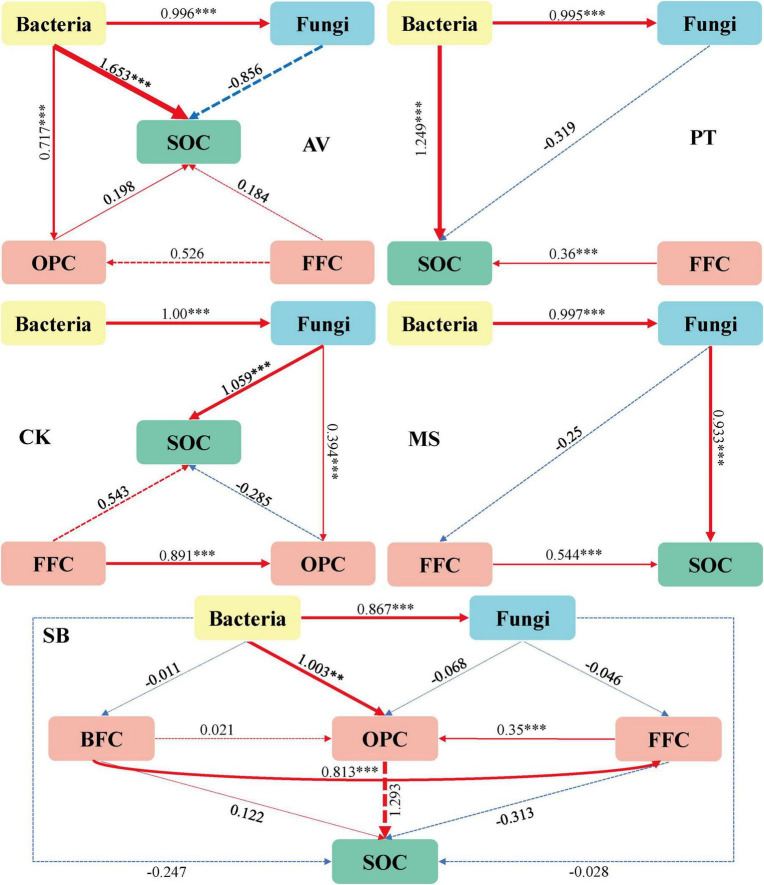
Path analysis model of the degradation pathway of the soil micro-food web under different vegetation types. *Prunus armeniaca* L. (AV), X^2^ = 0.165, df = 3, *p* = 0.983, CFI = 1.000, GFI = 0.991, RMSEA = 0.000, NFI = 0.998, TLI = 1.147; *Pinus tabuliformis* Carrière (PT), X^2^ = 0.294, df = 2, *p* = 0.863, CFI = 1.000, GFI = 0.977, RMSEA = 0.000, NFI = 0.996, TLI = 1.084; *Caragana korshinskii* (CK), X^2^ = 3.845, df = 4, *p* = 0.427, CFI = 1.000, GFI = 0.852, RMSEA = 0.000, NFI = 0.965, TLI = 1.004;*Medicago sativa* L. (MS), X^2^ = 0.388, df = 2, *p* = 0.824, CFI = 1.000, GFI = 0.970, RMSEA = 0.000, NFI = 0.992, TLI = 1.122;*Stipa bungeana* (SB), X^2^ = 0.066, df = 2, *p* = 0.967, CFI = 1.000, GFI = 0.997, RMSEA = 0.000, NFI = 0.999, TLI = 1.192. The width of the arrows is proportional to the strength of the path coefficients. The red and blue arrows denote positive and negative relationships, respectively, while the solid and dashed lines represent significant and non-significant relationships. BFC, the carbon metabolic footprint of bacterial feeders; FFC, the carbon metabolic footprint of fungal feeders; OPC, the carbon metabolic footprint of omnivores-predators; SOC, soil organic carbon. **P ≤ 0.01, ***P ≤ 0.001.

As illustrated in Figures 2A–E, the soil microbial and nematode networks of *C. korshinskii* exhibited the highest number of connections, followed by *M. sativa*, *P. tabuliformis*, and *S. bungeana*, with *P. armeniaca* displaying the fewest connections. Additionally, all treatments had more positive connections than negative ones (Table 1). In *S. bungeana*, *P. tabuliformis*, and *M. sativa* treatments, bacteria primarily drove the degradation channel in the soil micro-food web. In *P. armeniaca*, fungi were the primary drivers. In *C. korshinskii*, bacteria, and fungi influenced the soil micro-food web (Figures 2F–J). Furthermore, the analysis of carbon flow in the soil micro-food web (Figure 3) showed that fungi contributed the most to soil organic carbon in *C. korshinskii*. In contrast, bacteria contributed the most to soil organic carbon in *P. armeniaca*.

**TABLE 1:** Related network parameters for different vegetation types.

Index	*Prunus armeniaca* L.	*Pinus tabuliformis* Carrière	*Caragana korshinskii*	*Medicago sativa L.*	*Stipa bungeana*
Number of nodes	40	77	74	77	67
Total links	68	118	129	119	110
Positive links	44	75	75	74	82
Negative links	24	43	54	45	28
Average degree	3.40	3.07	3.49	3.09	3.28
Average node size	9.80	7.95	7.76	7.32	7.28

### Analysis of the metabolic footprint of soil nematodes, energy flow within the food web, and the functional index of the soil nematode community

**FIGURE 4:**
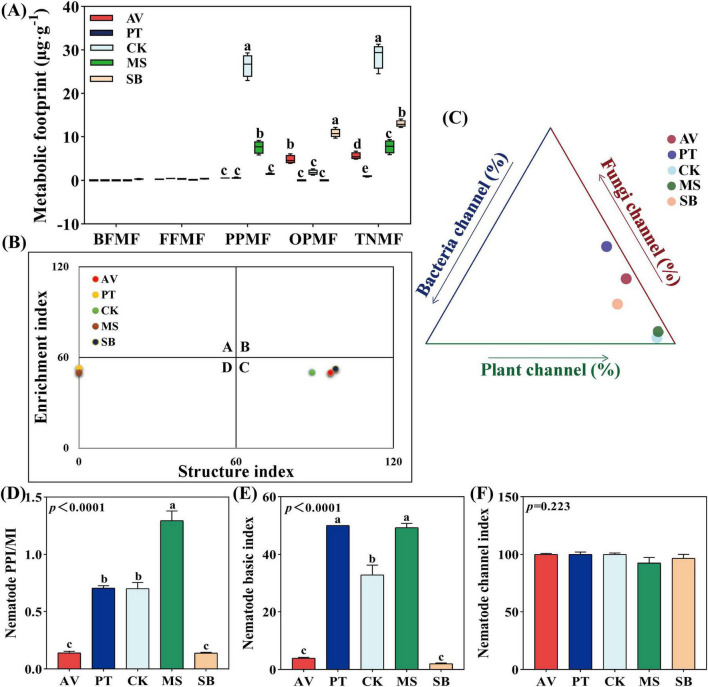
Metabolic footprint, floristic analysis, food web energy flow analysis, and functional structure index of soil nematodes under diverse vegetation types. **(A)** Metabolic footprint; **(B)** flora analysis; **(C)** food web energy flow analysis; **(D–F)** functional structural indices. AV, *Prunus armeniaca* L.; PT, *Pinus tabuliformis* Carrière; CK, *Caragana korshinskii*; MS, *Medicago sativa* L.; SB, *Stipa bungeana*. BFMF, The metabolic footprint of bacterial feeders; FFMF, The metabolic footprint of fungal feeders; PPMF, The metabolic footprint of plant-parasites; OPMF, The metabolic footprint of omnivores-predators; TNMF, The metabolic footprint of total nematodes. PPI, plant-parasitic nematode maturity index; MI, maturity index. Different lowercase letters indicate significant differences at *P* < 0.05 based on one-way ANOVA.

The plant parasitic nematode metabolic footprint and the total nematode metabolic footprint were highest in *C. korshinskii*, at 26.44 μg⋅g^–1^ and 28.65 μg⋅g^–1^, respectively, which were 18 times and 2.2 times higher than those in *S. bungeana*. The omnivorous-predatory nematode metabolic footprint was 0 μg⋅g^–1^ in both *P. tabuliformis* and *M. sativa* (Figure 4A). The metabolic footprint-based nematode faunal analysis (Figure 4B) showed that *C. korshinskii*, *P. armeniaca*, and *S. bungeana* were located in quadrant C, whereas *P. tabuliformis* and *M. sativa* were located in quadrant D. The food web energy flow analysis (Figure 4C) showed that *C. korshinskii* had the highest proportion in the plant energy flow channel (98.68%), which was 46% greater than that of *S. bungeana*. Furthermore, the ratio of the plant-parasitic index to the maturity index of *M. sativa* and the basic indices of *P. tabuliformis* (50) and *M. sativa* (49.25) were significantly higher than those of other vegetation types (*P* < 0.05) and the channel index of all five vegetation types exceeded 50 (Figures 4D–F).

### The interrelationship among soil nematodes, soil microbial communities, and environmental factors

**FIGURE 5:**
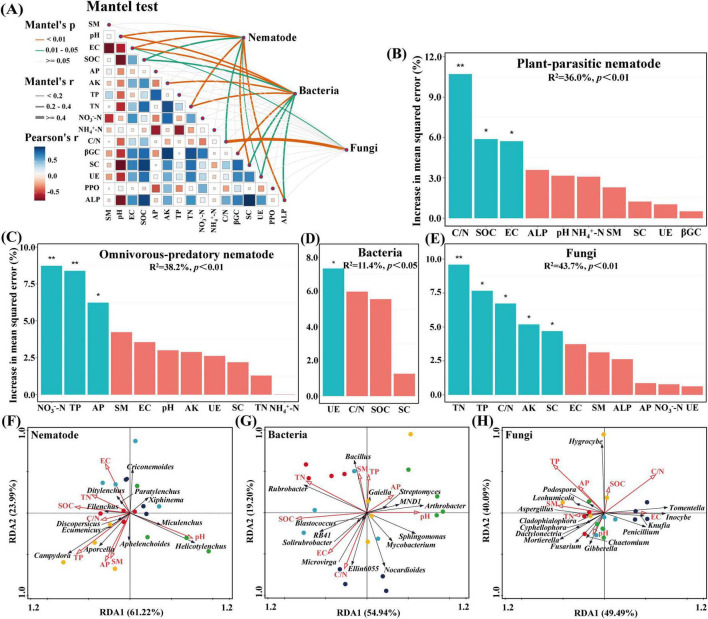
Factors influencing soil microorganisms and nematode communities. **(A)** The Mantel test disclosing the correlations between soil microorganisms and nematode communities, soil physicochemical properties, and soil enzymes. **(B–E)** Random Forest analysis exploring the explanatory factors of plant-parasitic and omnivores-predatory nematodes, bacteria, and fungi. Significance levels were indicated by *P < 0.05, **P < 0.01. **(F–H)** Redundancy analysis of the soil nematode, bacterial, and fungal community concerning environmental factors. SM, EC, SOC, AP, AK, TP, TN, NO_3_^–^-N, NH_4_^+^-N, C/N, βGC, SC, UE, PPO, and ALP, respectively, correspond to soil moisture, electric conductivity, soil organic carbon, available phosphorus, available potassium, total phosphorus, total nitrogen, nitrate nitrogen, ammonium nitrogen, the ratio of carbon to nitrogen, β-1, 4-glucosidase, sucrase, urease, polyphenol oxidase and alkaline phosphatases.

The composition of soil nematodes and microbial communities was significantly correlated with pH, electrical conductivity, total nitrogen, sucrase, alkaline phosphatases, soil organic carbon, the ratio of carbon to nitrogen, and urease (0.01 < *P* < 0.05) (Figure 5A and Supplementary Figure 1). The ratios of carbon to nitrogen, soil organic carbon, and electrical conductivity were the primary factors influencing plant-parasitic nematodes, while nitrate nitrogen, total phosphorus, and available phosphorus were the primary factors for omnivorous-predatory nematodes. Urease was the primary factor for bacteria (*P* < 0.05), and total nitrogen, total phosphorus, the ratio of carbon to nitrogen, available potassium, and sucrase were the primary factors for fungi (Figures 5B–E). Redundancy analysis (RDA) showed that environmental factors explained 85.21% of the total variance in soil nematode community abundance (RDA1: 61.22%, RDA2: 23.99%), with pH and total phosphorus significantly affecting the community composition (*P* < 0.05) (Figure 5F). For soil bacteria, environmental factors explained 74.14% of the total variance (RDA1: 54.94%, RDA2: 19.20%), with soil organic carbon being the primary factor (*P* < 0.05) (Figure 5G). For soil fungi, environmental factors explained 89.58% of the total variance (RDA1: 49.49%, RDA2: 40.09%), with total phosphorus and the ratio of carbon to nitrogen having the most significant effects (*P* < 0.05) (Figure 5H).

### Soil multifunctionality

**FIGURE 6:**
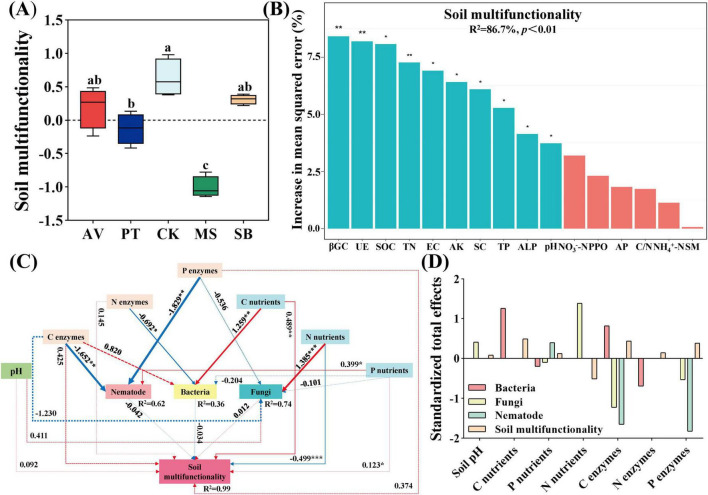
Factors influencing soil multifunctionality. **(A)** Alterations of soil multifunctionality across diverse vegetation types. Lowercase letters denote significant differences among treatments at *p* < 0.05. **(B)** Random forest analysis exploring the explanatory factors of soil multifunctionality. AV, *Prunus armeniaca* L.; PT, *Pinus tabuliformis* Carrière; CK, *Caragana korshinskii*; MS, *Medicago sativa* L.; SB, *Stipa bungeana*. βGC, UE, SOC, TN, EC, AK, SC, TP, ALP, NO_3_^–^-N, PPO, AP, C/N, NH_4_^+^-N, and SM, respectively correspond to β-1, 4-glucosidase, urease, soil organic carbon, total nitrogen, electric conductivity, available potassium, sucrase, total phosphorus, alkaline phosphatases, nitrate nitrogen, polyphenol oxidase, available phosphorus, the ratio of carbon to nitrogen, ammonium nitrogen and soil moisture. **(C)** The structural equation model depicting the connections among soil physicochemical factors, microorganisms, and nematode communities, as well as enzymatic properties (X^2^ = 4.461, df = 12, *P* = 0.974, CFI = 1.000, GFI = 1.000, RMSEA = 0.000, NFI = 0.999, TLI = 1.125). Positive and negative paths are marked with red and blue arrows, respectively. In contrast, significant (marked by *P < 0.05, **P < 0.01, ***P < 0.001) and non-significant links were represented by solid and dashed arrows, respectively. The width of the lines represents the standardized regression weights. *R*^2^ values adjacent to the variables indicate the proportion of variance explained by the other variables. **(D)** Standardized total effects of soil physicochemical properties and the activity of enzymes associated with C, N, and P cycling derived from the model. C nutrients, P nutrients, N nutrients, C enzymes, N enzymes, and P enzymes correspond to carbon cycling nutrients, phosphorus cycling nutrients, nitrogen cycling nutrients, nitrogen cycling enzymes, and phosphorus cycling enzymes.

As illustrated in Figure 6A, compared with *S. bungeana*, *C. korshinskii* enhances soil multifunctionality, whereas *M. sativa* exhibits a significant reduction (*P* < 0.05). In addition, the key variables for predicting soil multifunctionality included β-1,4-glucosidase, urease, soil organic carbon, total nitrogen, electrical conductivity, available potassium, sucrase, total phosphorus, alkaline phosphatases, and pH (Figure 6B). Soil pH and carbon cycling nutrients exhibited an extremely significant positive correlation with soil multifunctionality (*P* < 0.01). Nitrogen-cycling enzymes, phosphorus-cycling enzymes, and nitrogen-cycling nutrients showed a significant positive correlation (*P* < 0.05), while phosphorus-cycling nutrients had an extremely significant negative correlation (*P* < 0.01). Soil bacteria also exhibited a significant negative correlation (*P* < 0.05) (Figure 6C). Nutrients related to carbon cycling (0.487) and phosphorus cycling (0.124), as well as enzyme activities (0.440 and 0.382) were the primary positive effect factors influencing the structure of the soil micro-food web and the soil multifunctionality. In contrast, nutrients related to nitrogen cycling (-0.516) were the primary negative effect factors (Figure 6D).

## Discussion

### The influence of vegetation types on the soil micro-food web and the metabolic footprint of soil nematodes

Vegetation types differ in their root exudates, litter composition, and decomposition rates, influencing soil water and organic carbon distributions. These changes affect the composition and diversity of soil microbial and nematode communities, shaping the structure of the soil micro-food web (Lavelle, 1997; Johnson et al., 2003; Cesarz et al., 2013; Cai et al., 2022; Sha et al., 2023). Following artificial forestation in karst-degraded areas, the soil nematode community diversity index increased, and the nematode food web developed (Hu et al., 2016), which is consistent with this study. This study revealed that bacteria were the primary drivers of the degradation channel in the soil micro-food web in *S. bungeana*, *P. tabuliformis*, and *M. sativa*. At the same time, fungi were the dominant drivers of *P. armeniaca*. In *C. korshinskii*, both bacteria and fungi influenced the soil micro-food web, indicating that *C. korshinskii* and *P. armeniaca* have a greater capacity to protect carbon pools (Prescott and Vesterdal, 2021). Therefore, establishing *C. korshinskii* and *P. armeniaca* in Loess hilly areas not only facilitates the regeneration of understory herbaceous vegetation but also significantly improves soil structure and nutrient content, thereby enriching the soil with a considerable number of usable resources (Guan et al., 2015). However, as plantation age increases, the plant communities of *C. korshinskii* shrub forests degrade, reducing soil organic carbon, total nitrogen, and soil moisture, thus limiting the development of soil nematode communities (Li et al., 2015). In contrast, *P. armeniaca* arbor forest, characterized by favorable stand attributes and effective interception of rainfall through leaf gaps, mitigates soil erosion, enhances soil nutrients, and improves the structure of the soil micro-food web. Therefore, when restoring vegetation by planting *C. korshinskii* shrub forests in the region, it is imperative to establish appropriate *P. armeniaca* arbor forests on gentle slopes to safeguard the carbon pool.

The characteristics of soil nematode metabolic footprints provide effective methods and indicators for enhancing the study of soil multifunctionality by analyzing the carbon metabolic functions of different nematode groups and energy pathways within the soil micro-food web. This study revealed that the plant-parasitic nematode metabolic footprint and total nematode metabolic footprint of *C. korshinskii* were significantly higher than those of other vegetation types. This result indicates that the *C. korshinskii* shrub forests enhanced the metabolic activity of plant-parasitic and composite channels in soil nematodes. Given that plant-parasitic nematodes can effectively promote the allocation of photosynthate from plants to the rhizosphere, establishing artificial *C. korshinskii* shrub forests can not only enhance plant root exudates and microbial activity but also improve plant productivity (Ferris et al., 2001; Pan et al., 2021; Ruan et al., 2021).

Furthermore, metabolic footprint-based nematode faunal analysis revealed that *C. korshinskii*, *P. armeniaca*, and *S. bungeana* were located in quadrant C. This result contributed to maintaining soil ecosystem stability and enhancing the connectivity and complexity of soil micro-food webs. Conversely, *P. tabuliformis* and *M. sativa* are situated in quadrant D, exacerbating the degree of soil micro-food web disturbance in the area, leading to severe depletion of soil nutrients and structural degradation of soil micro-food webs. Studies by Cui et al. (2020) and Cai et al. (2022) also demonstrated that the soil micro-food web structure of shrubs and grassland vegetation types is more stable. However, *P. tabuliformis*, characterized by deep roots and strong allelopathic effects, is better suited for growth in fertile soil and deep soil layers. However, the soil in the study area was classified as yellow loess soil, characterized by its loose structure, low nutrient content, and severe erosion. Consequently, establishing *P. tabuliformis* in this region exacerbates soil erosion, leading to a decline in soil nutrients and a reduction in the diversity of soil microorganisms and nematodes. This, in turn, would seriously impede plant root exudation and microbial activity, thereby aggravating ecological degradation. In this study, the alfalfa meadow selected for *M. sativa* treatment had only been established for two years, resulting in low soil nematode metabolic activity, plant productivity, and significant soil micro-food web structure degradation. These findings differ from those reported in other studies (Hu et al., 2017).

### The influence of different vegetation types on soil multifunctionality

Different vegetation types influence soil physical and chemical properties as well as the structure of soil micro-food webs (Maestre et al., 2012; Zhang R. et al., 2021), thereby affecting soil multifunctionality (Aponte et al., 2013; Wang et al., 2022; Xu et al., 2022). Han et al. (2022) and Li et al. (2022a) reported that bacterial diversity and community composition in subtropical forests and fungal diversity in northern forests are the primary drivers of soil multifunctionality, respectively. This study found that *C. korshinskii* and *P. armeniaca* significantly promoted soil multifunctionality compared to *S. bungeana*. Enzyme activities related to carbon metabolism (β-1,4-glucosidase, sucrase) and nitrogen metabolism (urease) were high in *C. korshinskii* and low in *P. tabuliformis* and *M. sativa*. This is because the stand characteristics and leaf gaps of *P. tabuliformis* hinder its ability to effectively intercept rainfall, leading to severe damage to the soil structure, reduced soil permeability, and increased soil degradation. Additionally, this results in substantial mortality of surface vegetation, significant loss of organic matter from both the soil surface and interior, and a reduction in the input of soil organic matter at the source (Agarwal, 1999). Consequently, this lowers the stability of soil micro-food webs and harms soil multifunctionality (Li et al., 2022b).

**FIGURE 7:**
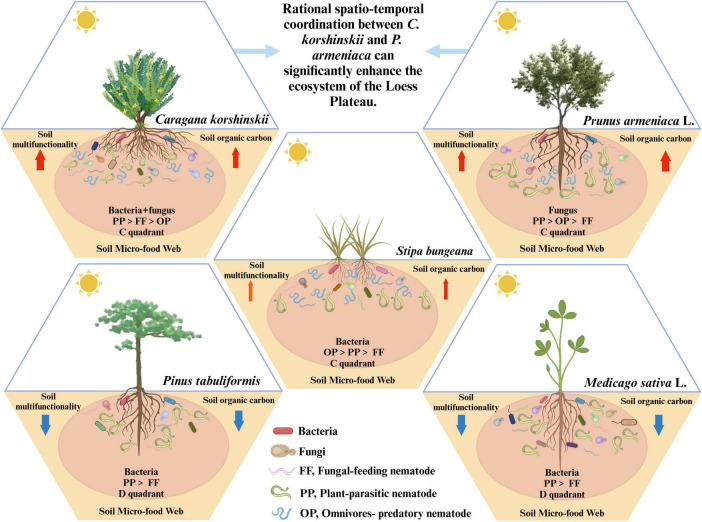
The contribution of arbor, shrub, and grassland types to the structural stability of the soil micro-food web and soil multifunctionality.

Therefore, establishing *C. korshinskii* can effectively mitigate soil erosion and enhance soil organic matter input in regions characterized by poor soil quality and severe soil erosion, such as Loess hilly areas. This restores soil nutrients, maintains the stability of soil micro-food web structures, and promotes soil multifunctionality (Figure 7). Additionally, *P. armeniaca* not only exhibits traits of drought resistance, tolerance to poor soils, and salt-alkali resistance but also produces litter that can be efficiently decomposed and absorbed by the soil, contributing to increased storage of soil organic carbon. Consequently, to better enhance soil multifunctionality, the appropriate integration of *C. korshinskii* and *P. armeniaca* is essential.

As the diversity of vegetation increases, the total rate of photosynthesis and the total carbon pool in the ecosystem also increase (Hu et al., 2017), leading to faster and more intense nutrient cycling, thereby promoting material transformation in the soil ecosystem (Zhang C. et al., 2016; Cui et al., 2020). Therefore, in regions with severe ecological degradation, such as Loess hilly areas, relying solely on a single type of vegetation for ecological restoration is insufficient. A comprehensive system comprising arbors, shrubs, and grasslands must be established and appropriately configured in space and time to effectively protect the ecological environment. The findings of this study can aid in predicting the responses of soil micro-food webs and soil multifunctionality to different vegetation types in plant production systems, thereby facilitating the identification of the most suitable vegetation types for specific areas and promoting the enhancement of the ecological environment.

## Conclusion

In the Loess hilly region, establishing *C. korshinskii* shrub forests and *P. armeniaca* L. arbor forests has demonstrated significant benefits in maintaining soil nutrients, stabilizing soil micro-food web structures, and enhancing soil multifunctionality. Notably, *C. korshinskii* shrub forests exhibited superior performance in these areas. Therefore, a vegetation restoration strategy that prioritizes the establishment of artificial *C. korshinskii* shrub forests, supplemented by artificial *P. armeniaca* arbor forests, represents an effective approach to improving the ecological environment in the loess hilly region.

## Data Availability

The data presented in the study are deposited in the NCBI repository, accession numbers PRJNA1215674, PRJNA1215675, PRJNA1215676.
