# Supplementary material for: Vegetation types shape the soil micro-food web compositions and soil multifunctionality in Loess Plateau
Source: Front Microbiol. 2025 Feb 12;16:1523811. doi: 10.3389/fmicb.2025.1523811 (PMC11861372; doi:10.3389/fmicb.2025.1523811)
Supplement: Supplementary file 1 [file Data_Sheet_1.docx]

**Supplementary file**


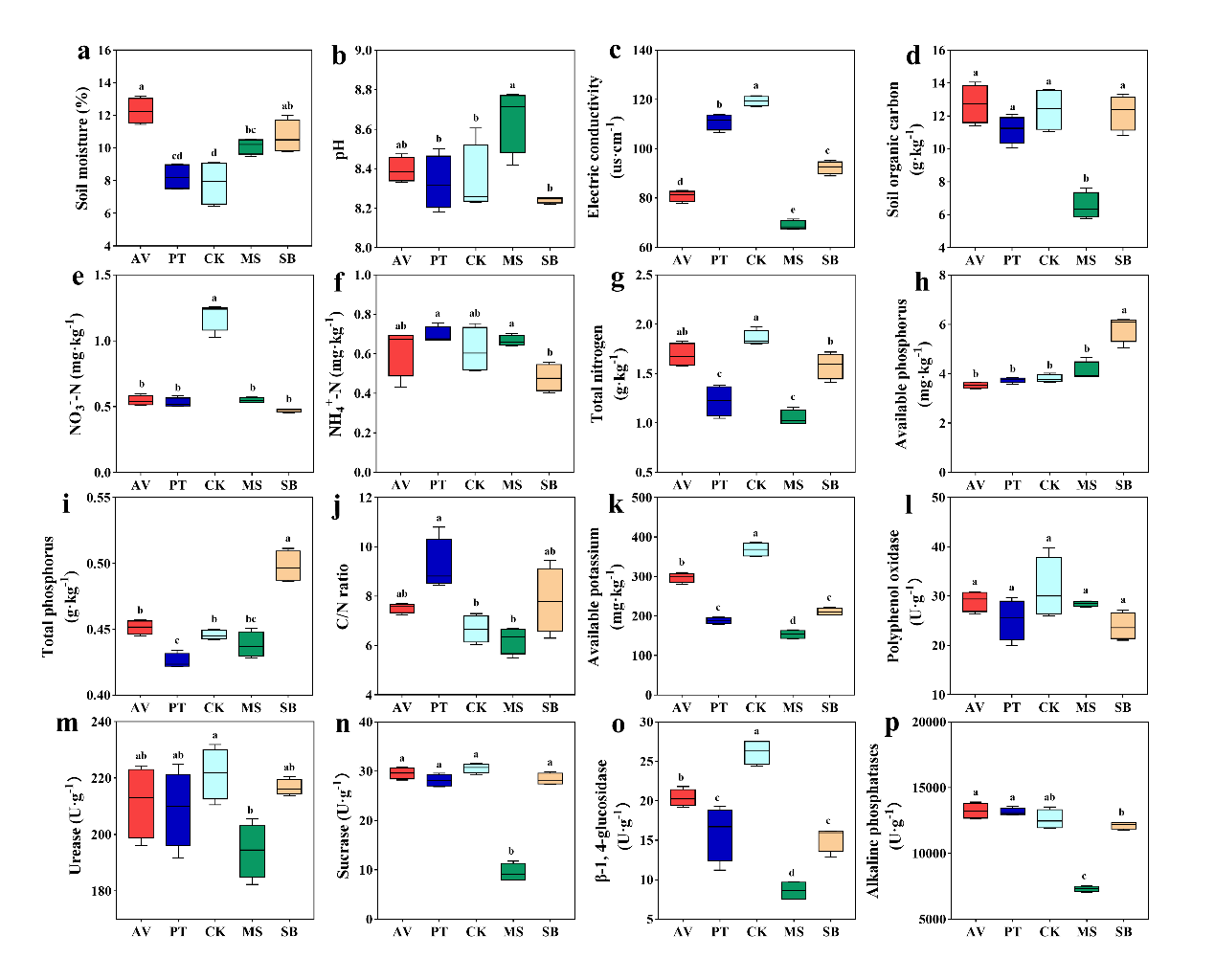


**Supplementary figure 1 Soil physical and chemical properties under different vegetation restoration treatments**. Values are mean ± SE in quadruple replicates. Different lowercase letters indicate significant differences with a value of *p* < 0.05 based on the ANOVA. C/N, Ratio of carbon to nitrogen.
